# Pubic pediculosis under the armpits of a homosexual male

**DOI:** 10.1590/0037-8682-0144-2024

**Published:** 2024-07-29

**Authors:** Huilin Zhi, Zehu Liu, Xiujiao Xia

**Affiliations:** 1 Hangzhou Third People’s Hospital, Department of Dermatology, Hangzhou Third Hospital Affiliated to Zhejiang Chinese Medical University, West Lake Rd 38, Hangzhou 310009, China

**FIGURE 1:**
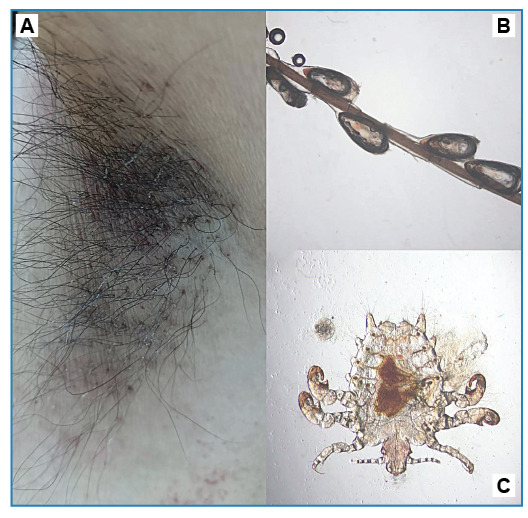
Clinical appearance of pubic lice infestation on the armpit area **(A)**. Numerous nits attached to hair shaft from the armpit area, visualized under microscopy (×20 magnification) **(B)**. Pubic lice morphology (×10 magnification) **(C)**.

A 55-year-old man who identified as homosexual visited our dermatology clinic and reported a persistent and severe itching sensation in the inguinal area and armpits over the past three months. He disclosed a history of homosexual contact and had received a diagnosis of perianal condyloma acuminatum one month prior. Physical examination revealed redness and black spots in both the groin and armpit areas, along with whitish concretions attached to the hair shafts (Figure 1A) and slow-moving parasites. Microscopy and dermoscopy revealed numerous nits glued to the hair on the armpit areas (Figure 1B), and direct microscopic examination of the armpit scraps revealed crab-like parasites (Figure 1C). We treated the patient and the sexual partner with a compound sulfur cream and instructed them to disinfect their clothes. One week after the treatment, the itching had resolved, and no lice were observed upon a follow-up examination at two weeks.

Lice are obligate parasites that feed only on the blood of infested hosts^1^. There are more than 3000 species of lice. Among them, only *Pediculus humanus* and *Phthirus pubis* (pubic lice) require humans as hosts. While pubic lice usually inhabit the hair of the pubic area, they can occasionally infest various hairy regions of the body, including under the armpits, in the beard or mustache, and on the eyebrows and eyelashes^2^. It is important to note that pubic lice are usually transmitted sexually and often coexist with other sexually transmitted diseases^3^, as in our case.
